# Supplementary material for: Design and Validation of a Gene-Targeted, Next-Generation Sequencing Panel for Routine Diagnosis in Gliomas
Source: Cancers (Basel). 2019 Jun 4;11(6):773. doi: 10.3390/cancers11060773 (PMC6627812; doi:10.3390/cancers11060773)
Supplement: Supplementary file 1 [file cancers-11-00773-s001.pdf]

cancers

MDPI

Supplementary Material

Design and Validation of a Gene-Targeted, Next-Generation Sequencing Panel for Routine Diagnosis in Gliomas

Nicky D'Haene, Bárbara Meléndez, Oriane Blanchard, Nancy De Nève, Laetitia Lebrun, Claude Van Campenhout and Isabelle Salmon

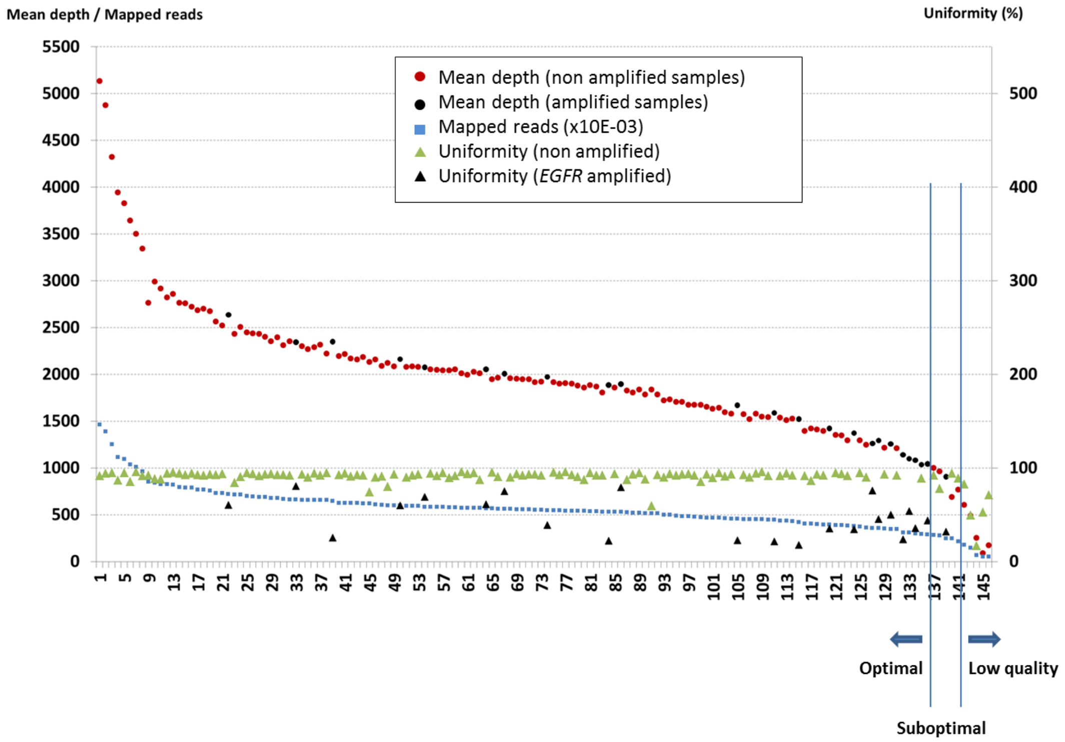

Figure S1. Graphical representation, for each sample, of the mean depth, mapped reads, and uniformity obtained in the NGS assays. Squares indicate the number of mapped reads, dots indicate the mean depth, and triangles indicate percentage of uniformity (note that in order to represent two of the variables under the same scale, the real mapped read values are 1000 times more than what is specified in the y-axis). The different colors are used to distinguish samples with *EGFR* amplification (black).

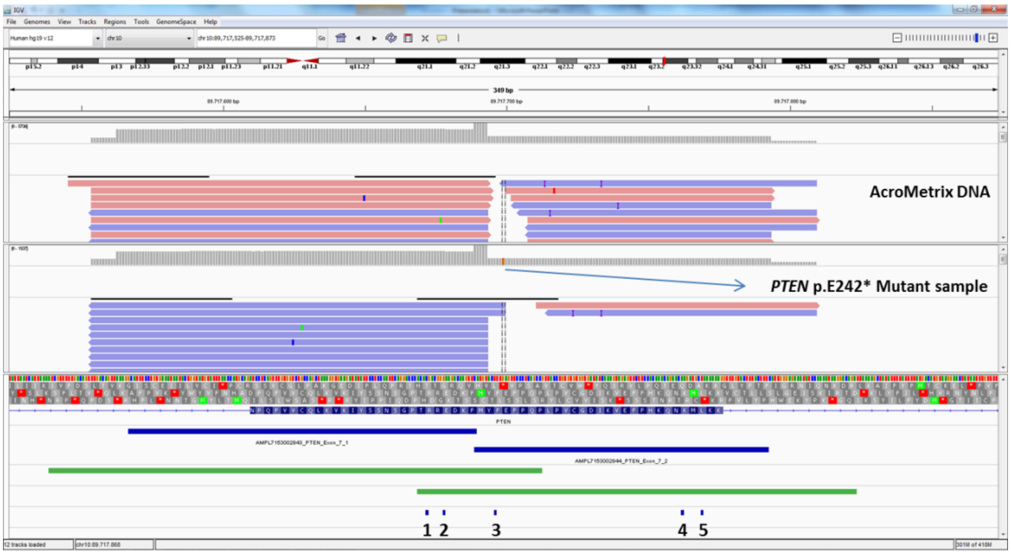

Figure S2. Visualization using Integrative Genomic Viewer of the AcroMetrix engineered DNA and a *PTEN* mutant sample. The green bars represent the length of the whole amplicons (insert and primers), while the blue bars above them represent the insert amplicons without the primers. The position of the described AcroMetrix mutations in the region are indicated by the blue squares and numbered 1 to 5. As shown, the variants 1 and 2 are located in the primer sequence of the right amplicon, and interfere with its amplification and the detection of variants 3, 4, and 5. Similarly, variant 3 is located in the primer sequence of the left amplicon, and impedes the amplification and detection of variants 1 and 2 that are located in the amplicon.

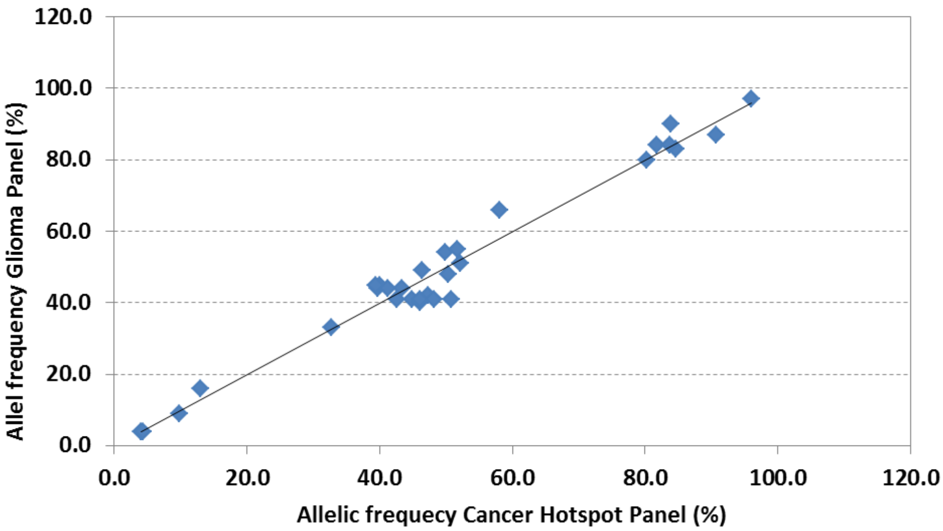

Figure S3. Comparative analyses of the allelic frequencies obtained with the cancer hotspot and glioma NGS panels. Allelic frequencies of 33 mutations from 22 glioma samples that were assessed for mutation detection with the CHP and glioma panels showed a high correlation between both panels (R = 0.99), even for those variants with an AF close to the limit of detection (4%).

Table S1. Validation of the NGS 1p/19q codeletion detection by fluorescence in situ hybridization (FISH).

NGS LOH Patterns	FISH Results				
	1p/19q Codeletion	No Codeletion	1p36 del	19q del	Total
1p/19q LOH	21	0	0	0	21
No LOH	1	18	1	0	20
LOH 1q	0	0	3	0	3
LOH 19q	0	0	0	5	5
Whole chr 1 LOH	0	2	0	0	2
Whole chr 19 LOH	0	6	1	0	7
Other patterns	0	2	0	0	2
Noninformative	0	3	1	1	5
Total	22	31	6	6	

Table S2. Summary of samples and patients included in the study.

		Samples			Patients		
Subtype	WHO Grade	Total	Validation	Prospective	Total	Validation	Prospective
Pilocytic	I	8	1	7	8	1	7
Oligodendroglioma IDH mutant,	II	15	11	4	14	10	4
1p/19q codeleted	III	10	6	4	9	6	3
Astrocytoma, IDH mutant	II	14	6	8	11	4	7
	III–IV	17	7	10	14	7	7
Astrocytoma, IDH wildtype	II	8	0	8	5	0	5
	III–IV	52	16	36	50	14	36
Glioblastoma-H3.3 K27M mutant	IV	5	3	2	5	3	2
Other diagnosis		10	1	9	9	1	8
Glioneuronal tumor		3	1	2	2	1	1
Subependymal giant cell astrocytoma		1	0	1	1	0	1
Tumor vs non tumor		5	0	5	5	0	5
Undifferentiated tumor		1	0	1	1	0	1
Non classified*		4	1	3	4	1	3
Total		143	52	91	129	47	82

*These cases could not be classified according to the 2016 WHO, due to inconclusive NGS results.

Table S3. SNPs used in the 1p/19q LOH analysis.

Chromosome arm	Start*	SNP	Chromosome arm	Start*	SNP
**Chr 1p**	16112795	rs7663	**Chr 19p**	751553	rs13345388
	19683301	rs169957		2720281	rs7256720
	23210600	rs309481		5063649	rs36115836
	26213991	rs159525		6818628	rs164020
	29245406	rs157208		8990761	rs57167556
	36168038	rs6425953		10265248	rs2114724
	40306898	rs7315		12505873	rs7246440
	45976472	rs7903		15120104	rs10419689
	53307957	rs504816		16842052	rs8107776
	55316322	rs7374		18108956	rs4808732
	60594980	rs87061	**Chr 19q**	30106659	rs7283
	65952428	rs11811946		31883906	rs2542297
	71477315	rs5680		34011248	rs33841
	76990862	rs191142		35615179	rs12852
	88776278	rs54396		38229378	rs1291
	91604522	rs106075		39926509	rs17628
	95394352	rs1132		40931717	rs166539
	101338324	rs8888		44090195	rs3817
	109289487	rs6604120		47112648	rs10113
	115110683	rs8128		48833800	rs8355
**Chr 1q**	156030820	rs2275073		51359497	rs11573
	162301446	rs347303		53073605	rs193040
	170198294	rs4575136		53611187	rs3814
	181286871	rs898114		56030428	rs10217
	189241655	rs1342566		59093239	rs10448
	195608036	rs12744553			
	204839154	rs2802849			
	210951585	rs1770214			
	219383192	rs6692892			
	228876199	rs16848862			
	156030820	rs2275073			
	162301446	rs347303			
	170198294	rs4575136			
	181286871	rs898114			
	189241655	rs1342566			

*Start position according to GRCh37 (hg19).
